# Extracellular vesicles subpopulation derived of clonal mesenchymal stem cells protected retinal ganglion cells in mouse model with optic nerve crush

**DOI:** 10.1016/j.reth.2026.101145

**Published:** 2026-06-06

**Authors:** Leila Satarian, Nasim Eslami, Rezvaneh Saeedi-fard, Elham Yektadoost, Amir Shojaei, Fatemeh Sadat Hosseini Mazinani, Leila Taghiyar, Sara Poosti, Abdolreza Nazari, Fatemeh Zarei, Majid Halvaie, Faezeh Shekari

**Affiliations:** a Department of Stem Cells and Developmental Biology, Cell Science Research Center, Royan Institute for Stem Cell Biology and Technology, ACECR, Tehran, Iran; b Department of Brain and Cognitive Sciences, Cell Science Research Center, Royan Institute for Stem Cell Biology and Technology, ACECR, Tehran, Iran; c Department of Physiology, Faculty of Medical Sciences, Tarbiat Modares University, Tehran, Iran; d Institute of Molecular Oncology and Stem Cell Biology (IMOS), Ulm University Hospital, Ulm, 89081, Germany

**Keywords:** Optic nerve injury, Clonal mesenchymal stromal cells, Extracellular vesicles, Retinal ganglion cells, AKT, PI3 kinase

## Abstract

**Introduction:**

Recent studies have shown that Mesenchymal stem cell-derived extracellular vesicles (MSC-EVs) have significant potential to protect retinal ganglion cells (RGCs) function. Here, EVs were obtained from various MSCs, including dental pulp MSCs, trabecular meshwork MSCs, and bone marrow-derived clonal MSCs (cMSCs).

**Methods:**

The different fractions of EVs were separated by high-speed (20K) and ultra-speed (110K) centrifugation. The functionality of EVs was then assessed in a mouse model of optic nerve crush (ONC).

**Results:**

The result showed that all EVs derived from various MSC sources could improve the optomotor response in ONC mice. Visual behavior and retrograde tracing of RGCs showed that both subpopulations of EVs derived from cMSCs were efficient and had a higher survival rate in Brn3a-positive RGCs. The ratio of *p*-AKT/AKT and p-PI3K/PI3K increased, and the expression of procaspase and caspase decreased in the retina and optic nerve following treatment with 20K subpopulation of cMSC-EVs.

**Conclusions:**

These results suggest that the increased restoration of visual behavior after cMSC-EV-20K treatment may be related to the protection of the RGCs by multi-trophic factors in EVs. Because the production of the EV-20K fraction is more accessible with fewer facilities, it has more capacity to be translated into the clinics for the repair of damaged optic nerves.

## List of abbreviations

RGCs: Retinal ganglion cells
ONC: Optic nerve crush
MSCs: Mesenchymal stromal cells
EVs: Extracellular vesicles
cMSC: Bone marrow-derived clonal MSCs
dMSCs: Dental pulp-derived MSCs
tMSCs: Trabecular meshwork-derived MSCs
MSC-EVs: MSC-derived EVs
cMSC-EVs: cMSC-derived EVs
dMSCs-EVs: dMSC-derived EVs
tMSC-EVs: tMSC-derived EVs
GMP: Good manufacturing practice
VEP: Visual evoked potential

## 1 Introduction

Most neurodegenerative disorders affecting the retina led to partial or complete irreversible vision loss due to a lack of self-regenerative capabilities and effective treatments. Since visual information is transmitted by axons of the retinal ganglion cells (RGCs), loss of the RGCs is directly correlated with vision loss. RGC degeneration is underpinned by complex molecular interactions that signal neural death, including factors such as inflammation, oxidative stress, mitochondrial dysfunction, and axonal transport issues, which are particularly highlighted in glaucoma, which is well studied [1]. The neuroprotective strategy for such a multifactorial disease must be comprehensive and involve multiple pathways. Numerous researchers are exploring ways to improve the recovery of degenerating RGCs [reviewed in Ref [2] despite the lack of clear mechanisms related to post-injury recovery.

Extracellular vesicles (EVs), which are secreted by cells, are recognized for their regenerative potential [3]. Due to their diverse RNA, protein, and lipid contents [4,5], they have a high potential as nanomedicines for regenerative therapies, particularly in the field of eye diseases [6]. Preclinical studies have shown efficacy of EVs in various ocular diseases such as glaucoma [7], retinal ischemia [8], retinal detachment [9], and optic neuropathy [10]. A clinical trial with EV transplantation is also available in the field of dry eye disease (https://clinicaltrials.gov; NCT04213248). These exciting results demonstrate the positive therapeutic effects of EVs in a wide range of eye diseases and highlighted where future studies should be conducted to identify the best cell source and to find standard EVs based on Good Manufacturing Practice (GMP) standards for clinical use [3].

Significantly, the highlighted studies demonstrate the efficacy of EVs primarily derived from various sources of mesenchymal stromal cell (MSC) [11]. The preference for MSC-derived EVs is likely due to their immunomodulatory potential, trophic factors, and specific miRNAs, which make them therapeutically useful.

We have demonstrated the therapeutic efficacy of EVs isolated from human embryonic stem cell-derived MSCs in an optic nerve injury model in mice [12]. Despite the substantial evidence to support the safety and efficacy of various sources of MSCs in ocular therapy, reliable, reproducible, and high-quality MSC sources are still to be developed. It is therefore important to have EVs from homogeneous MSCs that are compatible with GMP, such as those from bone marrow-derived clonal MSCs (cMSCs) [13]. Nevertheless, the therapeutic effect of cMSCs-derived EVs (cMSC-EVs) on retinal diseases, especially in the optic nerve, has not been studied. The aim of this study is therefore to investigate the efficacy of EVs derived from various sources of MSCs, including trabecular meshwork MSCs (tMSCs), dental pulp stem cells (dMSCs), and homogeneous cMSCs in the optic nerve crush (ONC) mouse model. Our focus is to identify the most appropriate source of MSC-EVs for therapeutic use in patients with optic neuropathy.

## 2 Results

### 2.1 EV characterization

**Fig. 1:**
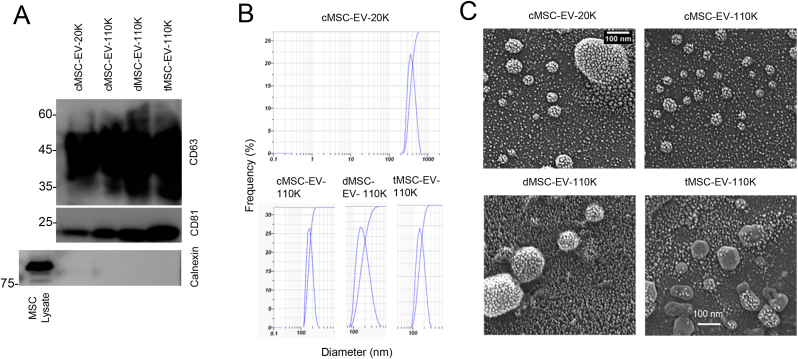
**Characterization of various sources of MSC-EVs.** (A) Western blot results for positive (CD63 and CD81) and negative (Calnexin) markers of EVs. This image is a cropped version of the entire blot, which is displayed in Supplementary Fig. 1 (B) Dynamic light scattering (DLS) of EVs. (C) Scanning electron microscopy images display the EVs (SEM; scale bar: 100 nm).

The presence of the typical EV markers, CD63 and CD81, and the absence of the negative marker, calnexin, in all EVs from different MSC sources was confirmed by Western blot analysis (Fig. 1A and Supplementary Fig. 1A). Dynamic light scattering measurements showed that EVs have variable size, with an average diameter of 360 nm for cMSC-EV-20K, 162 nm for cMSC-EV-110K, 178 nm for dMSC-EV-110K, and 155 nm for tMSC-EVs-110K (Fig. 1B). Scanning electron microscopy revealed that EVs had a spherical morphology (Fig. 1C).

### 2.2 MSC-EVs improved the visual behavior responses of optic nerve-injured mice

**Fig. 2:**
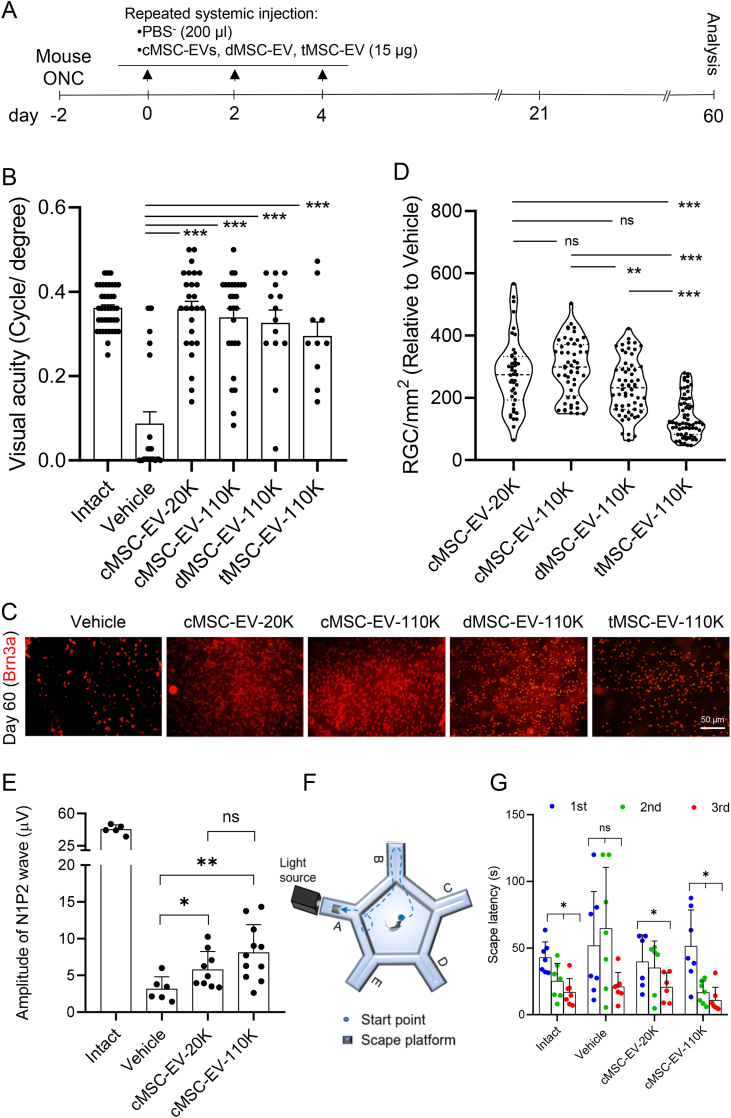
**Protective effect of EVs derived from different types of MSCs on damaged mice ganglion cells.** (A) A schematic representation of the experimental design. (B) Systemic injection of EVs derived from different types of MSCs in mice with optic nerve damage significantly improved visual acuity, as evaluated using optomotor response, 2-month post-injection compared to the vehicle group. There was no significant difference between different sources and subpopulations of EVs. (C) Immunostaining for Brn3a. (D) Quantification of retinal ganglion cell (RGC) survival, visualized by immunostaining for Brn3a. (E-G) Functional assessment of optic nerve repair. (E) Evaluation of changes in the amplitude of the N1P2 wave at 60 days post-injury in the vehicle group, cMSC-EV-20K, and 110K groups. Changes are expressed as the recovery in N1P2 amplitude post EV treatment. Significant recovery was observed in both EV groups compared to the vehicle group, with no significant difference between the two types of EVs. (F) A schematic representation of the modified 5-armed maze, indicating the start point, escape platform, and light source. (G) Time taken to reach the platform for the four experimental groups (intact, vehicle, cMSC-EV-20K, and 110K; n = 7/group, mean number of escape latencies from 3 trials for 3 days ±SD). Data are presented as mean ± SD and analyzed using one-way ANOVA with post-hoc Tukey's test, ∗∗P < 0.01 and ∗∗∗P < 0.001.

Two days after injury, mice were treated with intravenous injection of EVs derived from different MSC sources, as demonstrated in Fig. 2A. After two months, the functional analysis of the transplanted EVs was assessed by an optomotor response test, which evaluates visual acuity based on the animal's tracking of visual patterns displayed on the surrounding monitors. All EV-treated groups displayed significant improvement in response to visual stimuli (P < 0.001 compared to animals treated with vehicle, Fig. 2B).

Furthermore, survival rate of RGCs was assessed by expression of Brn3a, a transcription factor marker for RGCs, (Fig. 2C). Quantification of Brn3a-positive RGCs indicated that both cMSC-EV subpopulations had a higher density of RGCs than other MSC-EVs (at least P < 0.01, Fig. 2D). In the next step, the function of cMSC-EVs was assessed in ONC transplantation models of mice using VEP recordings.

VEP recordings, which gauge the functional integrity of the visual pathway up to the visual cortex were carried out to evaluate the impact of cMSC-EVs on axonal signal transport and the restoration of visual behavior 60 days post-injury. The amplitude of VEP waves, particularly the N1P2 wave, showed a high amplitude in healthy animals (baseline value = 42.5 ± 2.3 μV) and correlated with the number of axons transmitting signals. Although this wave's amplitude average value was significantly decreased in the vehicle group (= 3.1 ± 0.7 μV), it was considerably maintained in mice injected with both cMSC-EV subpopulations (= 5.7 ± 1.0 μV, P < 0.05 for cMSC-EV-20K and = 8.1 ± 1.5 μV, P < 0.01 for cMSC-EV-110K, Fig. 2E).

Furthermore, a behavioral test measuring light-avoidance, an innate response due to rodents' natural aversion to bright light [14], was implemented using a modified five-arm water maze. The time taken by mice to find an escape platform was recorded (Fig. 2F). Testing sessions were conducted daily for three days in intact mice, those treated with cMSC-EVs, and the vehicle group. On the second and third day, the vehicle group exhibited a significant increase in time taken to enter the arm with an illuminated platform, indicating impaired light-avoidance behavior (n = 7, P > 0.05). This increase was not observed in the healthy control or EV-treated groups (n = 7, P < 0.05, Fig. 2F), suggesting that both subpopulations of cMSC-EVs could restore light perception and avoidance behaviors. Therefore, visual behavior responses in ONC mice were improved more in both subpopulations of cMSC-EVs.

### 2.3 Capacity of EVs mediating neural survival via anti-apoptotic pathways

The isolation of cMSC-EV-20K requires less time and does not need more purification in comparison to cMSC-EV-110K. Furthermore, cMSC-EV-20K has more GMP compatibility and capacity for translation to the clinic due to efficiency and reproducibility in isolation for quality and in a scalable manner. Also, EVs have also been damaged by ultracentrifugation (110K) and this process has reduced the quality of the EVs [15]. Therefore, we explored the underlying mechanism of action of the injected cMSC-EV-20K for the restoration of optic nerve function.

**Fig. 3:**
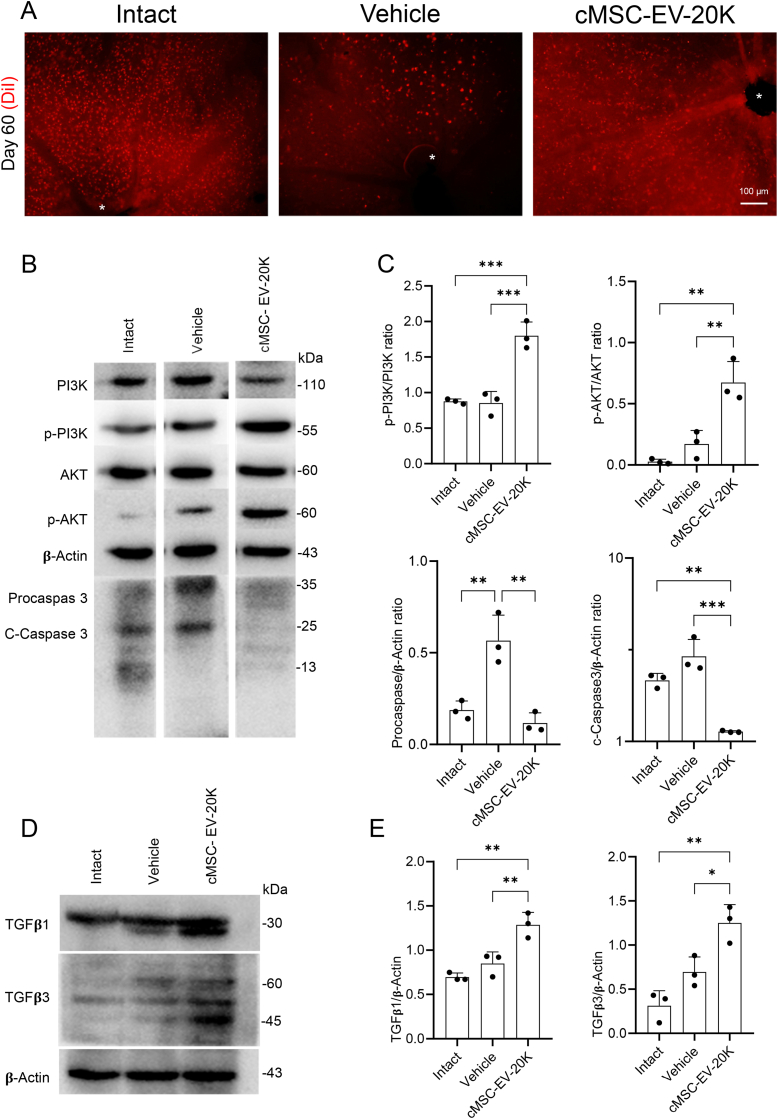
**Retrograde axonal tracing after EV treatment and Western blot analysis.** (A) Retrogradely-stained retinal ganglion cell soma in the intact, vehicle, and cMSC-EV-20K groups using DiIC18 (DiI; red). Each red dot represents a stained ganglion cell with an intact axon, with more stained cells observed in the treated group compared to the vehicle group. Stars demonstrated optic nerve head. (B) Western blot analysis of apoptosis factors from mice retina and optic nerves. (C) Densitometry of Western blots normalized to β-actin. (D) The expression of TGF-β3 and -β1, which are associated with ocular regeneration in the retina post-EV treatment. (E) Densitometry of TGF-β3 and -β1 Western blots normalized to β-actin. Data are presented as mean ± SD for three samples per group and analyzed using ANOVA with post hoc unpaired *t*-test. ∗∗P < 0.01, ∗∗∗P < 0.001.

Using retrograde tracing, we examined the whole-mount retinas of intact, vehicle-, and cMSC-EV-20K-treated eyes to determine the distribution of DiI throughout the RGCs. The uninterrupted DiI transport from the superior colliculus to the retina suggests maintained axonal integrity, which leads to the conclusion that cMSC-EV-20K has a protective effect on RGC axons two months post-injury (Fig. 3A).

Based on studies in apoptosis and axonal regeneration [16,17], we measured the activation (phosphorylation) of the PI3K/AKT pathway, which is pivotal for cell survival and axonal maintenance, by Western blot analysis (Fig. 3B). Quantification of phosphorylated AKT relative to total AKT and phosphorylated PI3K relative to PI3K was performed on the optic nerve and retina. Western blot analysis indicated a significantly higher proportion of *p*-AKT to AKT and p-PI3K to PI3K in the cMSC-EV-20K treated mice compared to those receiving vehicle (P < 0.01, Fig. 3C and Supplementary Fig. 2). Furthermore, the analysis of cleaved caspase-3 and procaspase activation was performed (Fig. 3B). Quantification of data demonstrated an increase of cleaved caspase-3 and procaspase in retina of the vehicle group, but no significant differences in *p*-AKT to AKT and p-PI3K to PI3K were observed in the cMSC-EV-20K group of mice and in healthy controls (at least P < 0.01, Fig. 3C and Supplementary Fig. 2).

Finally, we evaluated the expression of TGF-β3 and TGF-β1, which are associated with ocular regeneration, in the retina of EV-treated mice by Western blot analysis (Fig. 3D and Supplementary Fig. 3). Quantification of Western blot results demonstrated an increased levels of TGF-β3 and TGF-β1 expression in mice treated with cMSC-EV-20K (at least P < 0.5, Fig. 3E and Supplementary Fig. 3).

## 3 Discussion

Our study examined how EVs derived from various MSC sources can mitigate the pathological effects of ONC on RGCs. It is noteworthy that all EVs, especially both subpopulations of cMSC-EVs (20 K and 110K), were found to lead to improvements in visual behavior of the animals.

Recently, it has been reported that intravitreally transplantation of EVs derived from human-induced pluripotent stem cells (hiPSCs) and hiPSCs-differentiated neural progenitors in an ONC mouse model mitigated RGC degeneration and inhibited excessive activation of microglia through neuroprotective and anti-inflammatory effects [18].

In the field of ocular cell therapy, MSCs have been extensively studied in preclinical and clinical studies and are considered as experimental medicinal therapy. For more than two decades, MSCs from different sources such as umbilical cord, placenta, and adipose tissue, have been used in the treatment of ocular diseases such as traumatic optic neuropathies and diabetic neuropathy in animal and in clinical studies [[19], [20], [21]]. We observed the therapeutic efficacy of MSC-EVs in optic neuropathy, with notable anti-apoptotic, anti-inflammatory, and regenerative effects, as reflected by a significant increase in the number of GAP43-positive axons and cognitive visual behavior in ONC mouse models [12].

Despite the substantial evidence supporting the safety and efficacy of MSCs in preclinical and clinical studies [5], their efficacy is controversial, mainly due to the heterogeneity of the cell population. The establishment of homogeneous and high-quality MSCs could resolve these disparities and lead to consistent and reliable production. Here, we used a homogeneous population of cMSC produced by clonal selection under GMP standards [13].

At present, different approaches are used to separate the different subpopulations of EVs, but our understanding of their specific actions is still unclear [22,23]. Each subpopulation has different biological functions and therefore requires characterization [24,25]. Ultracentrifugation is the most common EV isolation method, known as EV-110K [26], but it is faced with significant challenges, such as low feasibility and the formation of large EV aggregates [27,28]. On the other hand, high-speed centrifugation, which isolates EV-20K, is easier and more practical for clinical use. Recently, we analyzed the proteomics of 20K- and 110K-EVs [29] and found that proteins involved in translation and metabolism are higher in 20K-EVs, while proteins involved in platelet activation, blood coagulation, and immune response are higher in 110K-EVs. Furthermore, we observed that the two subpopulations of EVs have comparable therapeutic effects. Consistent acetylcholinesterase enzyme activity was observed in both subpopulations, and a higher presence of apoptotic co-isolate components was reported in EV-20K [30]. The comparable functions of both subpopulations have been explored in animal models of chemotherapy-induced premature ovarian failure and Asherman syndrome [31,32]. Additionally, a phase 1 clinical trial demonstrated that co-administration of MSCs and their EV-20K significantly decreased the pro-inflammatory markers in COVID-19 patients without serious adverse events [33].

Considering that proteins may affect ocular regeneration, we reanalyzed our previous proteome report [29]. The proteomic reanalysis of cMSC-EV-20K and −110K revealed that the presence of factors such as HGF, IL-6, and TGF-β are associated with ocular regeneration in both EV types. However, FGF-2 and GAP-43 were unique to EV-20K, while NRG-1, NRGN, and BMP-1 were unique to EV-110K. These findings suggest that the MSC source and the isolation approach of EVs have an impact on the quantity, quality, and cargo composition of EVs.

In conclusion, our findings indicate that cMSC-EV-20K improved visual function and promoted the survival of RGCs and axonal support in the retina of the ONC mice model. The feasibility and cost-effectiveness of cMSC-EV-20K according to GMP standards have the potential to support the RGCs and optic nerve, to reduce pathology and to improve functional recovery.

## 4 Materials and methods

**Extracellular vesicle isolation and characterization.** EV extraction and analysis was carried out as follows: dMSCs were obtained from the Royan Stem Cell Bank (RSCB, Royan Institute, Tehran, Iran) and tMSCs were generously provided by the Zanjan University of Medical Sciences [34]. cMSC cultured by GMP standards were obtained from the ATMP center of the Royan Institute. These cells were confirmed to have spindle-shaped and uniform morphology, standard markers for MSCs, and multiple potential for differentiation into skeletal lineage.

Both subpopulations of EVs, 110K and 20K were isolated by ultracentrifugation at 110,000 g and high-speed centrifugation at 20,000 g, respectively, as previously described from 48 h-conditioned media [31].

Characterization was made based on the MISEV2018 and MISEV2023 guidelines [35,36].

**Animals.** Male C57BL/J6 mice, approximately 8-10 weeks of age were used in the study. These mice were housed and treated in accordance with the guidelines of Royan Institute's Animal Use and Care Committee.

**Experimental design.** In this experimental setting, a group of mice of similar ages served as a control group and model groups were randomly assigned to five different groups after induction of ONC on day 2. One group of mice then received 200 μl of PBS as vehicle control, and other groups received systemic injections of various MSC-derived EVs, including cMSC-EV-20K, cMSC-EV-110K, dMSC-EV-110K, and tMSC-EV-110K, each at 15 μg per dose in a total of 200 μl PBS. The injections were given every other day, three times post-crush. The concentration of EVs was determined using the bicinchoninic acid protein assay (Thermo Fisher Scientific).

**Optic nerve crush (ONC).** Anesthesia was induced in animals with 1:4 mixture of Xylazine/Ketamine. Intra-orbital ONC was performed as previously described [12]. In summary, the optic nerve was carefully exposed just below the superior edge of the orbit and then compressed for 5 s with precision fine forceps, specifically with #5B forceps (from World Precision Instruments), placed 1 mm apart from the eyeball to ensure that the retinal artery was not ruptured during the compression.

**Visual behavior test (Optomotor response).** Visual behavior tests were conducted 60 days after ONC to assess optomotor response. The spatial visual acuity of mice was measured using the OptoDrum device (ThorLabs, Germany). During the test, each mouse was first examined and then placed on an elevated platform surrounded by a virtual cylinder consisting of four screens, with the center of the cylinder aligned with the mouse's head.

The spatial frequency of the left or right eye was independently evaluated by looking at the direction of the grating movement of the monitor, either clockwise or counter-clockwise [37]. Initially, the gratings were set to a low spatial frequency. Mice that could detect movement instinctively follow the stripes with corresponding movements of their heads. The frequency gradually increased until the animal's visual threshold was surpassed, indicated by the cessation of the optomotor reflex. This method established the mouse's visual acuity, which was quantified in cycles per degree.

**Water maze**. The water maze task was conducted using a custom-built opaque Plexiglas maze with five corridors stemming from a pentagonal central area, each corridor being 10 cm wide, 14.5 cm long, and 22 cm high. The central area had an apothem (the shortest distance from the center to any side) of 33.4 cm [14]. The pool was filled with water up to the level of escape platform, which was 6.5 cm wide and 12 cm tall. An LED guide light emitting at a wavelength of 385 nm was positioned at the entrance of the corridor containing the platform. Each mouse was released into the maze's center and observed for 120 s to see if it could find and climb onto the escape platform, thereby completing the trial. This test was conducted over three consecutive days, including the first, second, and third trials, to account for the learning effect. The time of mounting to the platform was recorded, with two researchers, who were not informed of the experimental conditions, timing the trials.

**Visual evoked potential (VEP) recording.** VEP recordings were taken before ONC induction and again at 60 days after treatment in mice. The VEP procedure was conducted according to the established protocol [38]. The amplitude of N1P2 wave was measured from each VEP trace and used to assess amplitude recovery, with a sample size of 5 to 10 eyes per group.

**Tissue preparation.** 60 days after ONC procedure, animals were euthanized by lethal CO_2_ overdose. Immediately thereafter, the retinae and optic nerves were isolated, rinsed in PBS, and stored at −80 °C for the Western blot test. Some mice were perfused with saline intracardially followed by 4% paraformaldehyde to obtain tissue fixation. In the retrograde tracing studies, the eyes were enucleated and post-fixed in 4% PFA for an additional 2 h, after which the retinae were carefully removed and placed on slides for imaging purposes. The eyes designated for the flat mount technique were stored in PBS at 4 °C post-perfusion.

**Retinal flat mount imaging for RGC survival analysis**. To quantify the survival of RGCs in different groups, retinal flat mounts were immunostained as previously described [12]. The count of Brn3a positive cells (16 images from each retina; 4 eyes per group) was performed manually by an observer who was not informed of the allocation of the groups.

**Retrograde tracing.** Retrograde tracing studies were conducted to assess the neuronal pathways between the eyes and the brain in mice 60 days after treatments. DiIC18 (DiI, 2 μl) was injected into the superior colliculus of mice.

**Western blot analysis of apoptosis factors from mice retina and optic nerves.** The levels of the different apoptotic markers (phosphorylated PI3K (p-PI3K), PI3K, phosphorylated AKT (*p*-AKT), AKT, procaspase, caspase, TGFβ3, and TGFβ1) and EV protein markers, CD63 and CD81 (positive markers), and Calnexin (a negative organelle marker), were assessed by a Western blot. Optical density (OD) of the bands from the tissue blots was measured using ImageJ software and the OD values were normalized to the level of β-Actin levels. The list of antibodies is presented in Supplementary Table-1.

**Statistical analysis.** Statistical assessments were performed using ANOVA followed by Tukey's post hoc test or the unpaired *t*-test as appropriate. Results are presented as mean ± standard deviation (SD).

## Author contributions

LS contributed to conceptualization, methodology, validation, supervision, and writing (reviewing and editing); NE contributed to investigation and original draft preparation; RS contributed to investigation; AS contributed to validation; FH contributed to investigation and original draft preparation; LT contributed to validation; SP contributed to investigation; FS contributed to validation, supervision, and writing; AN contributed to investigation; FZ contributed to investigation; MH contributed to methodology; EY contributed to investigation.

During the preparation of this work the authors used GPT-5 to Paraphrase. After using this service, the authors reviewed and edited the content as needed and took full responsibility for the content of the publication.

## Availability of data and material

All data generated or analyzed during this study are included in this published article.

## Ethical approval and consent to participate

All animal experiments were approved by the Research Ethics Committee of the Royan Institute, Tehran, Iran (IR.ACECR.AEC.1401.055, January 31, 2023) under the project title “To compare the effects of different fractions of clonal mesenchymal stromal cell derived extracellular vesicles on nerve repair in optic nerve crush mice model”.

This study also received ethical approval for human dental pulp stem cells from Research Ethics Committees of Royan Institute, Tehran, Iran (EC/90/1072, August 28, 2011) with the project title “Isolation, Characterization and Comparative Differentiation of Human Dental Pulp Stem Cells Derived from Permanent Teeth by Using Two Different Methods” and for human trabecular mesenchymal stromal cells from the Research Ethics Committees of Zanjan University of Medical Sciences, Zanjan, Iran (IR.ZUMS.REC.1397.320, January 22, 2019) with the project title “Encapsulation of miR-7 Transducted Trabecular Mesenchymal stromal Cells Using Microfluidic System with the Aim of Repairing Rat Spinal Cord Injury Contusion Model”. This study is reported in accordance with ARRIVE guidelines (https://arriveguidelines.org).

## Funding declaration

This study received support from the 10.13039/501100004920Royan Institute and the Iranian Research Association for Vision and Ophthalmology (IRAVO).

## Declaration of competing interest

The authors declare that they have no conflicts of interest with the contents of this article.
