# Supplementary material for: Extracellular vesicles subpopulation derived of clonal mesenchymal stem cells protected retinal ganglion cells in mouse model with optic nerve crush
Source: Regen Ther. 2026 Jun 6;33:101145. doi: 10.1016/j.reth.2026.101145 (PMC13264237; doi:10.1016/j.reth.2026.101145)
Supplement: Multimedia component 1 [file mmc1.docx]

**Supplementary Figure 1. Uncropped Western blots are related to Fig. 1A.**

**Supplementary Figure 2. The replicates of Western blots are related to Fig. 3B.**

**Supplementary Figure 3. The replicates of Western blots related to Fig. 3D.**

**Supplementary Table 1:** Antibodies used in this study.

| Antibody (Ab) | Company | Catalog no. | Dilution rate | |
| --- | --- | --- | --- | --- |
| Primary Ab |  |  | Immunostaining | Western blot |
| Brn3a | Santa Cruz | SC-31984 | 1:250 |  |
| CD63 | Santa Cruz | SC-5275 |  | 1:500 |
| CD81 | Santa Cruz | SC-7637 |  | 1:500 |
| Calnexin | Santa Cruz | SC-11397 |  | 1:500 |
| p-PI3K | Cell signaling Teq | 4228 |  | 1:1000 |
| PI3k | Cell signaling Teq | 4249 |  | 1:1000 |
| p-AKT | Cell signaling Teq | 4060 |  | 1:1000 |
| AKT | Cell signaling Teq | 4691 |  | 1:1000 |
| Cleaved caspase-3 | Cell signaling Teq. | 9661 |  | 1:1000 |
| TGFβ3 | Abcam | Ab15537 |  | 1:1000 |
| TGFβ1 | Abcam | Ab92486 |  | 1:1000 |
| β-actin | Proteintech | HRP-60008 |  | 1:20 000 |
| Secondary Ab |  |  |  |  |
| [Alexa Fluor® 568 Donkey Anti-goat (IgG)](https://www.abcam.com/Donkey-Goat-IgG-HL-Alexa-Fluor-568-preadsorbed-ab175704.html) | Abcam | Ab175704 | 1:500 |  |
| Rabbit anti-mouse IgG antibody, HRP conjugate | Sigma-Aldrich | AP160P |  | 1:100 000 |
| Goat anti-rabbit IgG antibody, HRP conjugate | Abcam | ab102287 |  | 1:50 000 |
