# Supplementary material for: Extracellular vesicles subpopulation derived of clonal mesenchymal stem cells protected retinal ganglion cells in mouse model with optic nerve crush
Source: Regen Ther. 2026 Jun 6;33:101145. doi: 10.1016/j.reth.2026.101145 (PMC13264237; doi:10.1016/j.reth.2026.101145)
Supplement: Multimedia component 2 [file mmc2.pptx]

## Slide 1
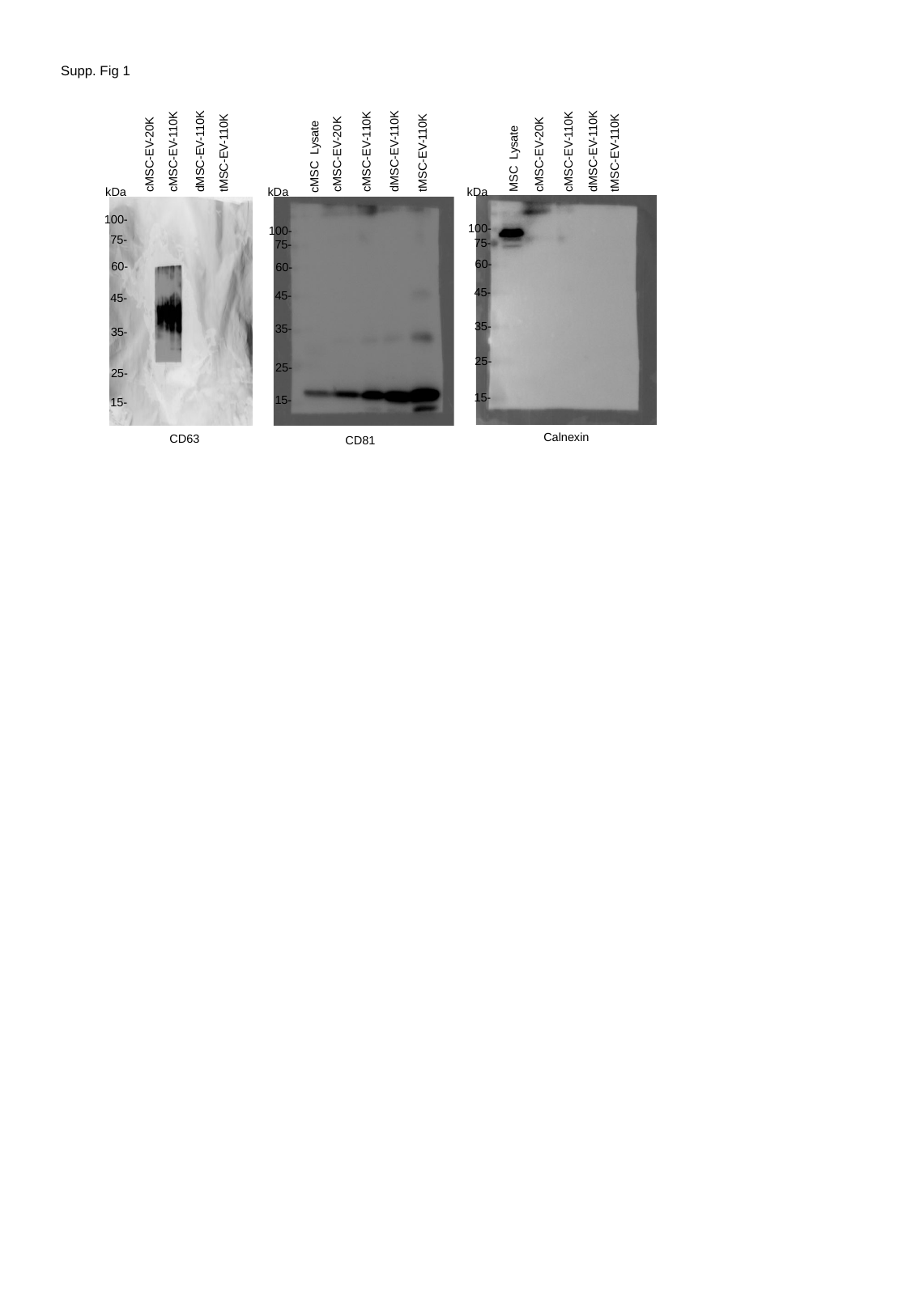

Supp. Fig 1
tMSC-EV-110K
cMSC-EV-20K
dMSC-EV-110K
cMSC-EV-110K
cMSC Lysate
100-
75-
60-
45-
35-
25-
15-
CD81
cMSC-EV-110K
tMSC-EV-110K
dMSC-EV-110K
MSC Lysate
cMSC-EV-20K
100-
75-
60-
45-
35-
25-
15-
Calnexin
cMSC-EV-20K
tMSC-EV-110K
dMSC-EV-110K
cMSC-EV-110K
kDa
kDa
kDa
100-
75-
60-
45-
35-
25-
15-
CD63

## Slide 2
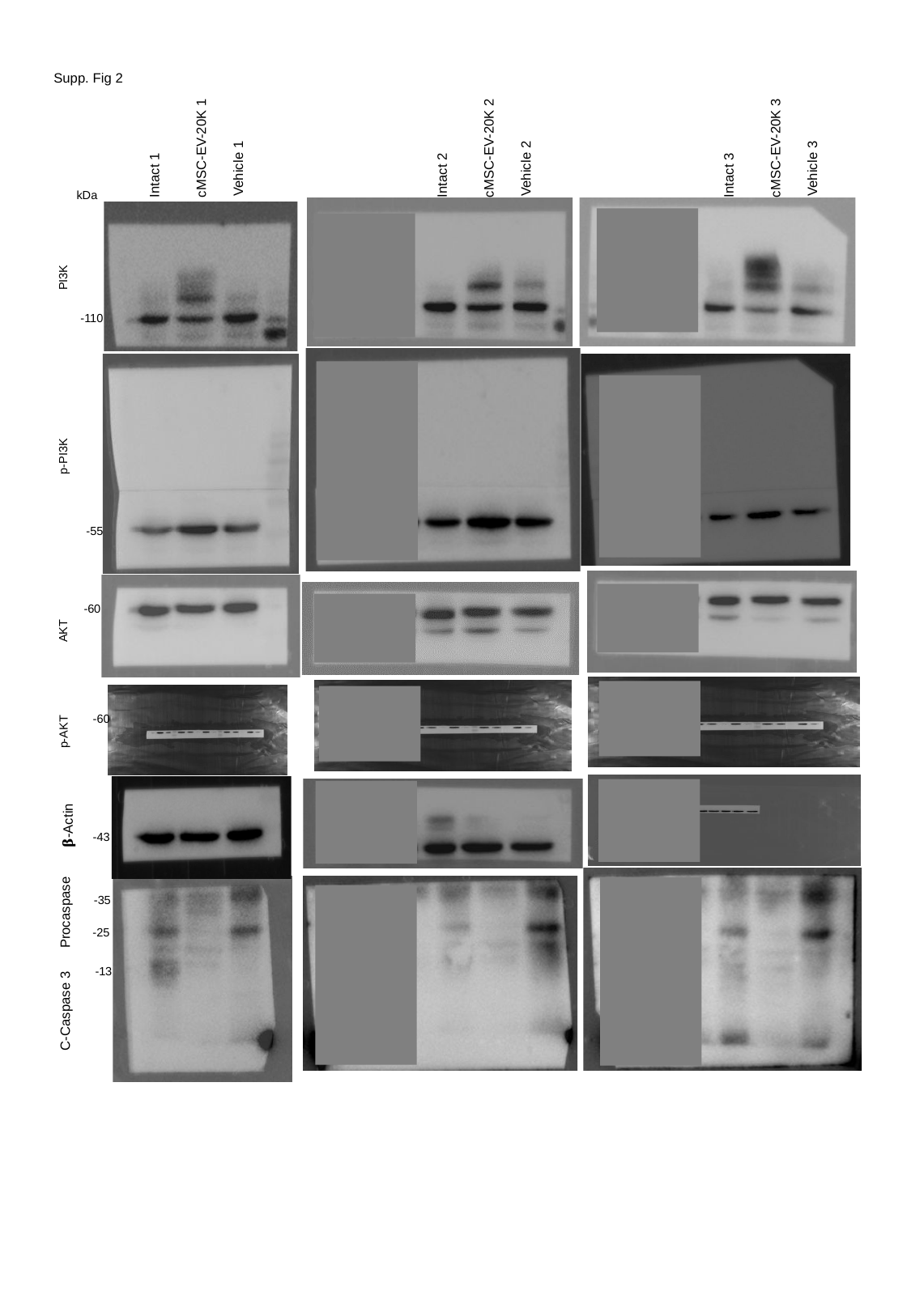

cMSC-EV-20K 2
Vehicle 2
Intact 2
cMSC-EV-20K 3
Vehicle 3
Intact 3
cMSC-EV-20K 1
Vehicle 1
Intact 1
Supp. Fig 2
kDa
PI3K
110-
p-PI3K
55-
60-
AKT
p-AKT
60-
𝛃-Actin
43-
35-
Procaspase
25-
13-
C-Caspase 3

## Slide 3
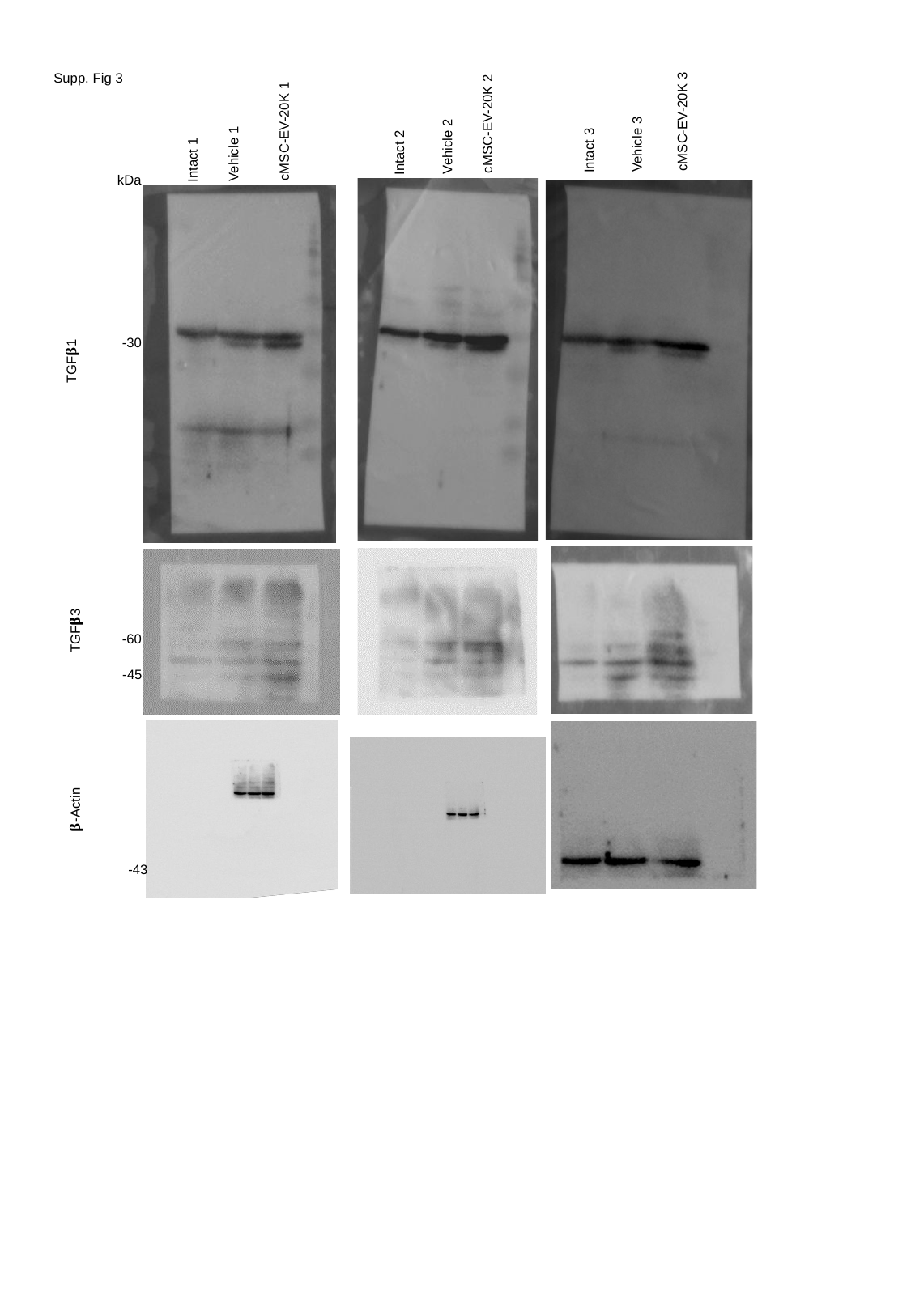

cMSC-EV-20K 1
Vehicle 1
Intact 1
cMSC-EV-20K 2
Vehicle 2
Intact 2
cMSC-EV-20K 3
Vehicle 3
Intact 3
kDa
30-
TGF𝛃1
TGF𝛃3
60-
45-
𝛃-Actin
43-
Supp. Fig 3
